# The Inevitable Relationship Between Viruses and RNA Modifications Revealed Through Adenovirus Research

**DOI:** 10.3390/v18020243

**Published:** 2026-02-14

**Authors:** Shuichi Hashimoto, Fumiaki Uchiumi, Hideaki Furuya, Radhakrishnan Padmanabhan

**Affiliations:** 1 Department of Gene Regulation, Faculty of Pharmaceutical Science, Tokyo University of Science, Niijyuku 6-3-1, Katsushika-ku, Tokyo 125-8585, Japan; 2 Department of Microbiology and Immunology, Georgetown University Medical Center, Washington, DC 20057, USA

**Keywords:** viral multiplication, RNA modification, adenovirus, methylation, transcription initiation site, alternative splicing, viral oncogenes

## Abstract

Over the past two decades, it has become clear that gene expression in eukaryotic cells is regulated by diverse RNA molecules. In this process, new RNAs have been discovered, and the roles of their modified molecules have been progressively elucidated. In this review, we first describe how RNA and its modifications function in virus-infected cells. We use adenovirus and several other viruses as models during the early stages of infection, which we believe determines the fate of infected cells. Next, we reviewed the process of identifying the early mRNA transcription initiation sites in adenovirus-infected cells. The results showed that the transcription initiation sites for the *E1* and *E4* mRNAs—known as adenovirus oncogenes—are highly complex. The same level of complexity in transcription initiation sites has been suggested for oncogenes in several other DNA tumor viruses, including SV40, polyomavirus, and papillomavirus. It is now understood that the transcription of the early adenovirus mRNA involves alternative splicing, rather than constitutive splicing, as we previously demonstrated. Furthermore, recent research indicates that the abnormal alternative splicing of intracellular mRNA may induce cellular carcinogenesis. Finally, we discuss whether alternative splicing plays a role in the carcinogenic effects of DNA tumor viruses, such as adenovirus. Additionally, we discuss that alternative splicing plays a crucial role in adenovirus replication.

## 1. Introduction

RNA modifications identified as important for viral replication may have been first discovered in RNA viruses, such as cytoplasmic polyhedrosis virus (CPV) mRNA [1], reovirus mRNA [2], and vesicular stomatitis virus (VSV) mRNA [3]. In addition, modifications of RNAs such as Molony murine leukemia virus (MMLV) [4], avian sarcoma virus (ASV) [5], and Rous sarcoma virus (RSV) RNA [6] have been demonstrated.

At the same time, modifications of DNA virus-encoded mRNAs have also been studied in relation to viral proliferation. The mRNA generated in vitro by cores of vaccinia virus nuclei were shown to contain two cap structures at the 5′ end [7]. In addition, the methylation of mRNAs in simian virus 40 (SV40) [8] and human adenovirus type 2 (HAdV-C2) [9,10,11] has been studied. However, research on modifications of RNA derived from DNA viruses appears to have undergone unique developments and content that differs from those of RNA viruses.

Thomas and Green demonstrated that adding the protein synthesis inhibitor cycloheximide (CHX) to adenovirus-infected cells distinctly separates the synthesis of viral mRNA into the early and late phases of infection [12]. Early adenoviral mRNA is transcribed in the nucleus and processed in the absence of protein synthesis; therefore, host cell enzymes must perform these functions. Therefore, studying early adenoviral mRNA synthesis enables us to learn about the cellular mechanisms that regulate both viral and cellular gene expression. Furthermore, CHX has been shown to increase the yield of early adenoviral mRNA [13]. It has also been found useful for analyzing *E1a*-*E1b* overlapping mRNA, which is expressed at low levels in adenovirus-infected cells. We will discuss this mRNA in more detail (Section 5.2). CHX blocks the translocation reaction of transfer RNA on ribosomes during protein synthesis in eucaryotic cells. However, the mechanism by which CHX increases the yield of adenovirus early mRNA and *E1a*-*E1b* overlapping mRNA remains unclear.

An important discovery has been made in the field of RNA modification. This discovery concerns the occurrence of splicing during the transcription and processing of adenovirus mRNA [14,15]. Today we know that splicing is a fundamental process in the synthesis of mRNA in eukaryotic cells. Furthermore, recent advances in alternative splicing research are expected to be incorporated into studies on virus proliferation and carcinogenesis. It is truly interesting to know that the functional mRNA with alternative splicing was first discovered in adenovirus transcription research about 40 years ago. This review will also address this topic. It has been shown that alternative splicing occurs in the *E1* gene of early adenovirus genes. *E1* is an important gene group responsible for the carcinogenic function of the adenovirus and for viral proliferation in infected cells. Research on adenovirus mRNA alternative splicing has recently seen several significant studies published. These will be discussed in Section 5.2.

Furthermore, mRNAs derived from the oncogenes of DNA tumor viruses, HAdV, SV40, mouse polyomavirus (MPyV), human papillomavirus (HPV) and hepatitis B virus (HBV) appear to exhibit common characteristics. These include numerous transcriptional start sites and the production of diverse mRNAs with different splicing patterns. Conversely, it has been suggested that alternative splicing occurs in transcription utilizing multiple transcriptions or polyadenylation start sites within genes [16]. In other words, it is thought that alternative splicing is likely to occur during the transcription of mRNA in the oncogenes of these viruses. Research on the 5′-end heterogeneity of SV40, MPyVs, and HPV oncogene-derived mRNA has been compiled [17,18]. Despite decades of extensive studies and recent reviews on research into the carcinogenic mechanisms of HPV oncogenes, there remains another hurdle to overcome [19].

However, the modification of RNA in eukaryotic cells, including the type, extent and position of modifications to the base sequence, is still subject to various limitations [20,21]. In this review, we take up the analysis of RNA modifications in virus-infected cells, focusing on methods of analyzing the overall picture of viral mRNA modifications and the 5′ terminal base sequence, as well as important findings obtained from these studies.

RNA, particularly mRNA, has been shown to undergo internal modifications, such as m6A, that play numerous important roles. Several reviews have summarized these findings [22,23,24,25,26]. However, it has also been demonstrated that many m6A functions are exerted in conjunction with other factors.

We identified another important consideration when obtaining mRNA from virus-infected cells. Polyribosomes are sites where proteins are synthesized, but they are also known to possess regulatory functions that ultimately control mRNA translation. Furthermore, recent analyses have confirmed that siRNA/miRNA accumulate on polyribosomes. There are very few reports of viral mRNA being obtained from polyribosomes in virus-infected cells. In Section 2.2, we discuss adenovirus mRNA obtained in this manner. Specifically, we describe the procedure of obtaining virus mRNA by first obtaining the polyribosome of the infected cell and then separating it into poly(A)^+^RNA and poly(A)^−^RNA. In this review, we focus on papers that follow this procedure [27,28,29] and address two issues. One is that 5S RNA, presumed to be associated with polysomes, is invariably obtained from the HAdV-C2 poly(A)^−^RNA fraction. Another is that the same HAdV-C2 poly(A)^−^RNA fraction likely contains molecules from which only the poly(A) sequence has been removed from poly(A)^+^RNA, as inferred from its fractionation pattern. One of these relates to the finding that small interfering RNA (siRNA), which is believed to be associated with mRNA stability and translational activity, is processed and transported to polyribosomes to exert its effect [30].

In this review, we would also like to touch on the significance of non-coding RNAs (ncRNAs) in cells infected with viruses. Although the siRNA is a type of ncRNA, research into the role of ncRNAs in virus-infected cells is an area likely to see advancement in the future. The significance of ncRNAs in living organisms was recently reviewed [31]. ncRNAs introduced in the above review are also presumed to play a fundamental role in the mechanism of viral intracellular proliferation.

## 2. Modifications of Adenovirus Early mRNA and Their Interplay in the Virus Life Cycle

### 2.1. The Importance of Capturing the Full Picture of mRNA and Pre-mRNA Modifications in a Single Experiment

The analysis of RNA modification began in the 1960s with the study of eukaryotic tRNAs. When seeking to understand the overall pattern of modified bases in a specific RNA, a straightforward method involves first digesting the RNA molecule with RNase T2 and then separating the resulting digest through ion exchange column chromatography. By gradually increasing the salt concentration of the eluent, oligonucleotides adsorbed onto the separation resins of the column can be eluted and separated based on their differing degrees of binding to the support resins. When analyzing extremely minute amounts of RNA, such as viral early mRNA, it is necessary to radioactively label the RNA during the cell culture stage using [^32^PO_4_] or [*methyl*-^3^H], for example. The components of each charge fraction separated in this manner can be further analyzed for qualitative and quantitative determination through separation and analysis using re-chromatography or thin-layer chromatography. We have recognized that this method is highly effective for comparing modifications between two RNAs.

When HAdV-C2-infected KB cells are cultured in the presence or absence of CHX, the accumulation of early mRNA within the cells is approximately 5–10 times higher in the former group at 6–8 h post-infection. Regarding poly(A)^+^RNA, the number of internal m6A molecules per HAdV-C2 early mRNA molecule averaged 2–3 molecules in the presence of CHX and 4–5 molecules in its absence [29,32]. This suggests that excessive internal m6A in adenoviral early mRNA may make viral mRNAs more susceptible to degradation. However, the content of the 5′-terminal Cap structure within the mRNA and the ratio of Cap1 to Cap2 showed no difference when comparing the mRNA of these two [29,32]. Furthermore, the same research group also compared the modification of Ad2 early mRNA and MMLV genomic RNA. As a result, it was found that one 5′-terminal cap1 structure per RNA molecule is synthesized in MMLV genomic RNA. In conclusion, this analytical method is particularly useful when comparing the constituently modified molecules of two distinct types of RNA molecules.

As one example, the ratio of RNA modification molecules obtained using this analytical method is summarized in Table 1. However, the most interesting aspect of these results appears to be the comparison between mRNA and pre-mRNA. This is because the results are thought to reflect the role of modification molecules in the processing from pre-mRNA to mRNA. Furthermore, for comparison purposes, the analysis results for pre-mRNA adjusted under the same conditions as mRNA would be more suitable for this objective. Here, the results from pre-mRNA studies (Hashimoto and Green, unpublished result), conducted concurrently with mRNA research [29,32], were provided by Hashimoto for this review and are shown in Table 1.

The radioactively labeled HAdV-C2-specific mRNAs and pre-mRNAs were digested with RNase T2 and then chromatographed alongside oligonucleotide markers on a DEAE-Sephadex column. The −2, −3, −4, −5, and −6 charged fractions, which contained mononucleotides (including modified mononucleotides), dinucleotides (NmpNp), trinucleotides (pNp), tetranucleotides (Cap1) and pentanucleotides (Cap2), respectively, were separated, as shown in Table 1. The Cap1/Cap2 molecular ratio is approximately 1 for mRNAs and 4–5 for pre-mRNAs. These results indicate that approximately 80% of Cap2 in Ad2 early mRNA is produced in the cytoplasm. The average nucleotide length of poly(A)^+^ RNA and poly(A)^−^RNA was calculated from the content of the 5′ terminal caps in each molecule. For Ad2 early mRNAs, these values are 1250 and 960, respectively. For pre-mRNAs, these values are 5300 and 2900, respectively (Table 1).

The amount of 2′-*O*-methylation (NmpNp) in Ad2 pre-mRNA is approximately four to five molecules per pre-mRNA molecule, regardless of whether it is poly(A)^+^ or poly(A)^−^. For Ad2 early mRNA, one to two NmpNp molecules were confirmed in poly(A)^−^mRNA but not in poly(A)^+^ mRNA. These results imply that Ad2 pre-mRNA contains an average of four to five 2′-*O*-methylated ribose molecules, believed to be located within base sequences that are removed during pre-mRNA processing. A review article published in 2024 states that it is still not possible to conclude whether internal NmpNp exists in eukaryotic mRNA [33]. Therefore, the results of Hashimoto and Green are in good agreement with the current state of research on internal NmpNp in eukaryotic mRNA [29,32].

### 2.2. Information Related to Virus Proliferation Is Possessed by Polyribosomes and Cap Structures

When HAdV-C2 early mRNA was purified from the polyribosomes of infected cells, it was found that approximately 10–20% of the total was poly(A)^−^RNA. An analysis of each mRNA through polyacrylamide gel electrophoresis suggests that poly(A)^−^RNA is not RNA in which the poly(A) sequence has been randomly destroyed, but rather RNA in which the poly(A) sequence has been selectively deleted [29,32]. This estimation is based on the average length of the nucleotide chains of poly(A)^−^RNA and the results of the polyacrylamide gel electrophoresis analysis of the molecular weight distribution of RNA.

Interestingly, an unknown 5S RNA(s) was reproducibly recovered in the polyacrylamide gel electrophoresis analysis of HAdV-C2 poly(A)^−^RNA. This 5S RNA was never obtained from HAdV-C2 poly(A)^+^ RNA [29]. The authors state that these results are consistent whether the infected cells are cultured in the presence or absence of CHX. An interesting related paper is worth mentioning here. Kim et al. obtained miRNAs from the polyribosomes of animal neuronal cells [34]. In their paper, they conclude that despite the identification of over 200 miRNAs in plants and animals, very few miRNA targets have been confirmed experimentally. Furthermore, it has been shown that adenovirus-associated RNAII-derived small RNAs are efficiently incorporated into the RNA-induced silencing complex and associated with polyribosomes [30]. In other words, it is possible that specific small RNAs that perform functions for specific mRNAs accumulate on polyribosomes. For these reasons, obtaining mRNA from polysomes is considered important when isolating it from virus-infected cells. However, research focusing on this aspect is still relatively scarce, even today.

Processing viral pre-mRNA in the nucleus of a cell infected with a virus is thought to be one of the most important processes for understanding viral replication. However, the available information on this topic is still very limited. Adenovirus may be a good system for investigating the processing mechanism of viral pre-mRNA from the cell nucleus to the cytoplasm. Among the modified nucleotides of mRNAs that are related to viral proliferation, the 5′-terminal cap [35] and internal m6A are particularly important (discussed below). The 5′-terminal cap structure also provides important information about the nucleotide sequence of mRNA transcription initiation [35]. pNp is a structure commonly found at the 5′ end of successfully processed RNA. Therefore, the pNp obtained in the HAdV-C2 early poly(A)^−^mRNA is probably a nucleotide derived from the 5′ end of the 5S RNA that is associated through hybridization with the HAdV-C2 poly(A)^−^mRNA.

As shown in Table 1, HAdV-C2 mRNA contains approximately equal amounts of Cap1 and Cap2 at the 5′ end. However, there are few reports on the presence of Cap2 at the 5′ end of viral mRNA or genomic RNA. The appearance of Cap2 on RNA may be rare. Recently, Inagaki et al. found that Cap2-type mRNA (mRNA with only Cap2 at the 5′ end) exhibited a 3- to 4-fold higher translational activity than Cap1-type mRNA (mRNA with only Cap1 at the 5′ end) [36]. Another role of Cap2 has been demonstrated: Cap2 methylation provides a novel strategy to distinguish viral RNA from self-RNA and control the activation of the innate immune response [37]. In the next section, we will discuss the effects of internal m6A in mRNA on viral proliferation using several examples of viral mRNA.

Finally, we must mention the relationship between RNA modification and the mammalian innate immune system. Kariko et al. found that modified RNA molecules could suppress the activation of mammalian innate immune systems by DNA and RNA via Toll-like receptors (TLRs) [38]. It is interesting to note that most of the HAdV-C2 early mRNA modifications likely suppress Toll-like receptor recognition systems [38].

## 3. Occurrence of Internal m6A in Viral mRNA, Viral Genomic RNA and Non-Coding RNA in Normal and Transformed Cells

Next, we focus on the role of internal m6A in viral mRNA and non-coding RNA, which has recently been suggested to play an important role in the intramolecular modification of eukaryotic RNA. It is thought that m6A is closely related to the life cycle of mRNA, as well as to viral infection, cell development, differentiation and pathogenesis. In other words, the functions of internal m6A in RNA are diverse. Research into the location of m6A in mRNA base sequences, as well as into the system that controls m6A methylation, has advanced significantly, as has research into the structure and function of m6A in mRNA. Several reviews have been compiled on the significance of m6A modification of mRNA [25] and the associated mechanisms [22]. Here, we discuss the relationship between viral propagation and m6A modification. Our findings suggest that m6A modification of viral mRNA and genomic RNA often facilitates viral proliferation by suppressing the innate immune response.

### 3.1. Early and Late Adenoviral mRNAs

Both adenovirus early and late mRNAs contain four to five internal m6A molecules per molecule on average [32,39]. However, when transcribed in the presence of CHX, the early mRNA contains two to three m6A molecules per molecule [29].

The location and function of the internal m6A in adenovirus early and late mRNA has recently been reported by Price et al. [40]. The synthesis of the intramolecular m6A portion of adenoviral mRNA requires the cellular methyltransferase complex METTL3. Price et al. constructed A549 cells lacking METTL3 expression, infected them with Ad5 and analyzed the synthesis of viral mRNA and protein [40]. The knockdown of METTL3 specifically affects late viral transcription by reducing its splicing efficiency. In contrast, early mRNA species, except for *E1b-19K* mRNA, were unaffected by METTL3 knockdown. They observed a reduction in the levels of the late viral proteins: the hexon, the penton and the fiber. In contrast, the early viral proteins were unaffected. Additionally, the depletion of the m6A writer was shown to specifically affect late viral transcription by reducing its splicing efficiency. Only the *E1b-19K* early mRNA appears to adopt the splicing mechanism of the late mRNA.

### 3.2. SV40

Both the early and late mRNA of the SV40 virus have m6A modifications. The authors precisely localized m6A, which is distributed at ten positions in the late mRNA and two positions in the early mRNA of the SV40 virus [41]. First, the authors engineered BSC40 cells, an SV40-propagating cell line, to increase the expression of the m6A reader YTHDF2. When this cell line was infected with SV40, the authors found that SV40 proliferation was significantly increased. They also compared the plaques produced on YTHDF2-high BSC40 cells with those produced on control BSC40 cells. The plaques produced on YTHDF2-high BSC40 cells were larger in both size and number. Additionally, the authors observed that the loss of METTL3 expression in infected cells resulted in a decreased expression of the SV40 structural gene VP1 during the replication process, significantly decreasing SV40 viral replication [42]. However, the loss of METTL3 expression in infected cells did not affect the pattern of late gene mRNA splicing. In conclusion, the authors argue that adding m6A enhancers to SV40 late mRNA through the action of the cell-derived reader protein YTHDF2 promotes the expression of viral structural genes and consequent viral replication.

### 3.3. Kaposi’s Sarcoma-Associated Herpesvirus (KSHV)

As mentioned above, the m6A modification of viral mRNA in adenovirus and SV40 is an example of how it promotes viral replication in infected cells. However, in KSHV, the m6A modification has an antiviral effect [43]. The authors of this paper discovered that, in the case of KSHV, the m6A modification of RNA in infected cells, along with the concomitant recruitment of YTHDC2 (a reader that regulates the m6A modification of mRNA), exerts an antiviral effect. Many viruses, including KSHV, have been shown to reduce intracellular mRNA levels in infected cells in a process known as “host shut-off”. During lytic KSHV infection, a major shift in total mRNA abundance is driven by the viral endoribonuclease SOX, which induces the decay of more than 70% of transcripts. Furthermore, the authors observed that the SOX resistance element introduced by YTHDC2 can resist multiple viral endonucleases, but not cellular endonucleases. This makes it a virus-specific RNase escape element. This suggests that m6A may contribute to the host’s efforts to regulate gene expression.

### 3.4. Non-Segmented Negative-Sense RNA Viruses (NNS RNA Viruses)

Non-segmented non-segmented negative-sense (NNS) RNA viruses comprise a diverse group of pathogens affecting humans, animals, and plants. NNS RNA viruses are divided into five families. These viruses share many strategies for viral replication, gene expression and the evasion of the innate immune system. Representative viruses in this group have a single-stranded negative-sense RNA genome, and the complementary positive-sense anti-genomic RNA and mRNA are transcribed from it. Both the genomic and anti-genomic RNA molecules have a triphosphate at their 5′ ends, and the mRNA transcribed from the genomic RNA is capped [44]. Lu et al. mapped m6A in genomic RNA, anti-genomic RNA and mRNA isolated from cells infected with vesicular stomatitis virus (VSV) and Sendai virus (SeV) [44]. They generated strains of VSV, SeV, and MeV—growing cells deficient in the METTL3 complex. The defective and parental cells were then infected with the viruses, and virion RNA was harvested from each cell line. This was then transfected into A549 cells to measure type I interferon (IFN) production. The results indicated that the ability of m6A-deficient virion RNA to induce IFN is conserved among NNS RNA viruses, and conversely that m6A modification of virion RNA prevents IFN induction. Additionally, the authors discovered that m6A-deficient viral RNA enhances several steps in the RIG-I signaling pathway, and that the action of the m6A-binding protein YTHDF2 (an “m6A reader”) is necessary to suppress type I IFN expression. In conclusion, NNS RNA viruses acquire an m6A modification in their genome and anti-genome that enables them to evade recognition by the host’s cytosolic RIG-I innate immune system.

### 3.5. Occurrence of m6A in ncRNAs

To date, the main known targets of internal m6A modification are mRNA, pre-mRNA, and genomic RNA. All these RNAs contain genetic code. However, it has recently become clear that m6A modifications in ncRNAs are also functionally important. The m6A modification of ncRNAs in carcinogenesis can be considered one of the cellular responses to abnormal events occurring in normal cells. m6A modification of ncRNAs occurs in the nucleus, is dynamic and reversible, and is regulated by “writers”, “erasers” and “readers”. An analysis of ncRNAs from various tumor cells has suggested that m6A modification plays an important role in cancer development [24,45].

Consistent with this notion, some types of ncRNA modification may also occur during the viral infection of cells. However, research into virus-infected cells has not yet progressed to a sufficient degree to shed light on the role of ncRNAs in the viral life cycle.

## 4. Regulation of Viral Gene Expression by Small RNAs and ncRNAs

It is difficult to discern consistent patterns in the relationship between viral replication and small RNA or ncRNA within infected cells. Below are examples of widely known viruses and their small RNA profiles within infected cells. In this review, the nomenclature for these small RNAs will follow that were used in the original papers.

For instance, in the case of herpes simplex virus type 1, miRNAs that are derived from viral genes are believed to regulate various biological events, including immunological systems [46]. In the case of the SV40 virus, siRNA derived from the viral gene acts on mRNA in the early stages of infection. It suppresses the expression of the large T antigen and reduces the efficiency with which SV40-infected cells are cleared by cytotoxic T lymphocytes [47]. The hepatitis C virus (HCV) is known to use cell-derived siRNA. It is known that miR-122 is abundantly synthesized in human liver cells. miR-122 has been reported to promote the proliferation of HCV RNA [48]. Furthermore, when a cell is infected with a virus, it has been reported that cellular miRNA is induced in the case of HIV-1 [49], PFV-1 [50] and VSV [51]. Interestingly, all these miRNAs act to inhibit the growth of viruses within their host cells.

Here, we will discuss the appearance and functions of small RNAs and non-coding RNAs (ncRNAs) in HAdV-infected human cells, focusing on two examples: one derived from viral genes and the other from host cell genes. It has become clear that many of the small non-coding RNAs (sncRNAs) play a crucial role in the replication of HAdV within human-derived cells. SncRNA refers to short non-coding RNA molecules approximately 20–30 nucleotides in length that play crucial roles in regulating various biological processes. These are VA-RNA and MLP-TSS-sRNA. None of these RNA molecules possess protein translation activity, and they are transcribed from adenovirus DNA. The first VA-RNA promotes adenovirus replication within cells, while MLP-TSS-sRNA inhibits viral replication. VA RNA was the first viral sncRNA discovered in 1966 in HAdV-C2-infected KB cells [52]. Most types of HAdV possess two genes, VA-RNAI and VA-RNAII, but VA-RNAI is the major gene in terms of expression levels. The size of these VA-RNA genes is nearly identical in HAdV, approximately 160 nucleotides in length [53].

In 2006, Sano et al. [54] and Aparicio et al. [55] showed that HAdV-C2 VA-RNAI is processed into functional interfering RNAs. Furthermore, Xu et al. demonstrated that VA-RNAII-derived small RNAs are processed by Dicer into small RNAs that are incorporated into the RNA-induced silencing complex (RISC) and associate with polyribosomes [30]. Specifically, VA-RNAI and VA-RNAII are cleaved into miRNA-like molecules, namely mivaRNAI and mivaRNAII. The structure and function of VA-RNAs, their transition into miRNA-like molecules, and their functions have been elucidated by numerous research findings. For more detailed information on these topics, please refer to the review [56].

Another unusual sncRNA was detected in HAdV-C37-infected cells [57]. Since the transcription start site (TSS) of this sncRNA is located at the major late mRNA transcription start site (MLP) of the adenovirus DNA, it is termed the MLP-TSS-sRNA. The RNA molecule is 31 nucleotides long and possesses a cap structure at its 5′-end. It exhibits the most prominent expression in HAdV-C37-infected cells, though a weak expression is also observed in cells infected with other adenovirus types. Unlike mivaRNAs, MLP-TSS-sRNA is generated via the non-canonical miRNA pathway. Furthermore, this sncRNA appears to function as a regulator of viral DNA replication within Ad-infected cells.

We previously discussed the possibility that miRNAs/siRNAs are present in polyribosomes during the early stages of adenovirus infection. Xu et al. found that small RNAs derived from VA-RNAII associate with polyribosomes during the late stages of adenovirus infection [30]. The authors argued that these RNAs may regulate gene expression by functioning as miRNAs. Traditionally, VA RNA was thought to appear in the late stages of adenovirus infection. However, Crisostomo et al. discovered the presence of VA-RNAII 2–3 h after viral infection using a highly sensitive digital PCR system [58]. The fate and biological significance of VA-RNAII are of great interest.

Conversely, the role of ncRNA in pre-mRNA splicing is becoming better understood [31]. As discussed in Section 5.3, in HAdV-F40-replicating cells (A549 cells and KB18 cells), the expression of *E1a*-*E1b*-cotranscript mRNA appears to be required for viral replication [59,60]. It has been suggested that the splicing of this mRNA is abnormal and does not follow the GT-AG rule [60,61]. Therefore, in the HAdV-F40-replicationg cells, HAdV-F40 may receive cell-derived U-snRNA that enables this abnormal splicing and HAdV-F40 propagation.

## 5. Alternative Splicing (AS) Has Been Observed in mRNA Derived from the Adenovirus Oncogene E1

To date, numerous molecular biological studies of DNA tumor viruses, including adenovirus, SV40, PyV, HPV and HBV, have been published. Examining the transcription of mRNA from the oncogenes of these viruses reveals commonalities, such as the presence of numerous TSS and diverse types of mRNA due to splicing. In this section, we will explore the complexity of the TSS for adenovirus early mRNA and consider its significance. We wish to emphasize that, when dealing with highly complex TSSs, such as those found in DNA tumor virus early mRNA, it is necessary to first analyze the mRNA 5′-cap before proceeding directly to analyses like RACE (Rapid Amplification of cDNA Ends). The reason, though widely known, is that obtaining cDNA for mRNA is difficult, resulting in incomplete 5′-termini. This is likely why research reports clearly identifying mRNA TSSs using these new methods are scarce [62]. Furthermore, Adiconis et al. also showed that special methods are required to determine the 5′-terminal base sequences of eukaryotic cell mRNA [63].

### 5.1. The Transcription Start Site (TSS) of Adenovirus Early mRNA Is Extremely Complex

Determining the 5′-terminal nucleotide sequences of early adenovirus mRNA species is challenging due to the limited amount of mRNA that can be isolated from virus-infected cells. Methods to locate the 5′ end employed include nuclease gel analysis [64], electron microscopy [65], in vitro transcription initiation analysis [66] and primer extension analysis [67]. However, these methods cannot confirm the presence of a cap structure at the 5′ end. To determine the number of different 5′ ends, Hashimoto and his colleagues isolated, characterized and mapped the 5′-terminal oligonucleotides produced by the RNase T1 digestion of HAdV-C2 early mRNA from *E1* to *E4*.

Poly(A)^+^ polyribosome RNA was isolated from HAdV-C2-infected KB cells. The 5′ terminal m^7^Gppp was removed and the 5′ OH of the penultimate 2′-*O*-methylated nucleotide was labeled with [γ-^32^P] ATP using polynucleotide kinase [68,69]. Each transcription unit-specific 5′-^32^P-labeled mRNA preparation was isolated through hybridization to the Ad2 DNA restriction fragment. Each of these RNA preparations (*E1a*-, *E1b*-, *E2*-, *E3*-, and *E4*-specific 5′-^32^P-labeled mRNA) was digested with RNase T1; the resulting oligonucleotides were separated using the Sanger’s finger printing method [69,70,71,72,73].

### 5.2. E1a-E1b Cotranscripts Are Candidates for Alternative Splicing of mRNA and Are Found in mRNAs Transcribed from the E1 Region of Adenovirus Type 2, 5, 7, 12, and 40

The results of mRNA fingerprinting in the *E1* region (Table 2) are presented by Hashimoto et al. [74]. Table 2 reveals several intriguing details about the transcription start site of the *E1* region, particularly the presence of mRNAs that bridge the *E1a* and *E1b* regions. Spots 1 and 2 represent 5′ ends derived from the primary transcription start sites of *E1a* and *E1b* mRNAs, respectively [74].

Spots 1, a and d were detected in mRNA hybridized to the *E1a* region of DNA. Additionally, they were detected by mRNA hybridized to DNA from the *E1b* region. These results suggest that some of the mRNAs originating from the *E1a* region extend to the *E1b* region. This finding is consistent with the experimental results from the in vitro translation of mRNA [75]. Many reports have documented the production of *E1a-E1b* overlapping mRNAs in the *E1* region of adenoviruses.

The overlapping HAdV-C2 *E1a-E1b* mRNA in HAdV-C2 early infected cells was first reported by Buttner et al. [28]. Subsequently, a similar mRNA was detected in HAdV-C2-transformed cells [76] and in HAdV-C2-infected cells [64]. These overlapping mRNAs have also been reported in HAdV-B7-infected and -transformed cells [77], HAdV-A12-transformed cells [78,79] and HAdV-F40-infected A549 cells [59]. The translational activity of the *E1a-E1b* overlapping mRNA has also been reported [75]. Additionally, Maxfield and Spector discovered that the readthrough between the *E1a* and *E1b* genes is facilitated by the transcription of the *E1b* gene [80]. Recently, Price et al. reported the existence of *E1a-E1b* overlapping mRNA using a new long-read direct RNA sequencing method [81]. They also stated that this was accompanied through the discovery of novel viral splicing events and open reading frames. Does the synthesis of *E1a-E1b* overlapping mRNA benefit viral proliferation?

It has been reported that the E1a protein derived from the adenovirus type 2 gene causes necrosis and apoptosis in infected cells in the presence of TNF-α and anti-Fas antibodies [82]. They also found that cell death is suppressed by the HAdV-C2 E1b19K protein. In other words, the coexistence of the E1a and E1b 19K proteins is believed to be essential for adenovirus-infected cells.

As mentioned above, the synthesis of *E1a-E1b* overlapping mRNA has been the subject of much research. However, no studies have investigated whether this mRNA can be used as a template for protein synthesis. An analysis by Hashimoto et al. has suggested that at least three types of *E1a-E1b* mRNA exist, and that E1a cluster proteins, E1b 19K and pIX, are translated from 22S mRNA, which specifically hybridizes with *E1b* DNA [75]. It has also been shown that the E1b 19K protein is synthesized from mRNA that hybridizes with E1a encoding DNA. This paper presented the results of a more detailed analysis of the proteins derived from *E1a-E1b* mRNA. However, the precise splice sites in these mRNAs are not known.

Recently, the cDNA base sequences of HAdV-C2 early mRNA [83] and HAdV-C5 mRNA [84] were determined. The presence of alternative splicing and the splicing nucleotides were also identified. Furthermore, Westergren-Jakobsson et al. used long-read sequencing technology to determine the splicing position of HAdV-C2 early mRNA [85]. These results suggest that HAdV-C2 and HAdV-C5 early mRNA generate alternative splicing in addition to the previously reported patterns. However, some of these are detected at very low levels. In this review, we will discuss the alternative splicing of mRNA derived from oncogenes in DNA tumor viruses. Recently, it was found that alternative splicing in cellular gene expression related to cell proliferation leads to cancer [16].

It is known that the *E1a* and *E1b* genes, which are adenovirus oncogenes, encode proteins that are important for cell proliferation. However, the carcinogenic mechanism remains unclear. One of the most important aspects of adenovirus *E1* gene function is its association with tumorigenicity. The question, then, is whether the *E1a-E1b* overlapping mRNA is responsible for tumorigenicity. In this review, we introduce reports from two laboratories researching the tumorigenicity of adenoviruses. Both studies were conducted on the highly tumorigenic HAdV type 12, which belongs to group A. Saito et al. reported that the *E1a-E1b* overlapping mRNA (which they termed f.A-B1) was present in fully transformed rat cell lines infected with HAdV-A12, but not in incompletely transformed cell lines [79]. Furthermore, the expression of this mRNA was only detected during productive infection when CHX was added to the culture medium [79]. Mak and Mak reported that the transformation efficiency of primary rat kidney cells by HAdV-A12 *E1* region DNA fragments increased significantly when the transfected cultures were treated with CHX [86]. Furthermore, primary rat kidney cells can be efficiently transformed through CHX treatment using only 6.7% of the HAdV-A12 *E1* region DNA fragment from the left end. These transformed cells were also confirmed to be tumorigenic in animals. These results suggest the need for a significant revision of the conventional explanation of the carcinogenic mechanism of adenovirus oncogene *E1*. The need for the *E1b* region indicates that only the N-terminal part of the E1b 19K protein is necessary.

### 5.3. HAdV-F40: HAdV-F40 Propagation Is Enhanced Through the Synthesis of mRNA with Alternative Splicing in E1 Region

Many types of adenoviruses proliferate easily in KB or HeLa cells, resulting in a productive infection. However, HAdV-F40 does not proliferate in these cells (abortive infection). This issue has been studied for a long time, even in highly infectious pathogenic viruses, but remains unresolved. Mautner et al. demonstrated that HAdV-F40 can propagate in KB18 cells—a KB cell line containing Ad2 *E1b* genes—suggesting that Ad40 is defective in *E1b* gene function within KB and HeLa cells [60]. Hashimoto et al. demonstrated that HAdV-F40 can propagate in A549 cells (a human cell line derived from lung carcinoma that lacks *E1b* gene function) [59]. The analysis of HAdV-F40 mRNA in these cells strongly suggests that HAdV-F40 *E1b* mRNA and *E1a-E1b* overlapping mRNA beside *E1a* mRNA are synthesized in HAdV-F40-infected A549 cells during the early to intermediate stages after infection. Furthermore, Ishida et al. obtained multiple *E1a-E1b* overlapping mRNAs from HAdV-F40-infected A549 cells and determined the base sequence of their splicing junctions [61]. The sequence analysis revealed that the 5′ and 3′ splice junctions of the two *E1a-E1b* overlapping mRNAs did not conform to the GT-AT splicing consensus rule. This suggests that factors which lead to unusual splicing in the *E1* mRNA are present in HAdV-F40-infected A549 cells. Steinthorsdottir and Mautner found that HAdV-F40 *E1b* and *E1a-E1b* overlapping mRNAs beside *E1a* mRNA are synthesized in KB18 cells, which exhibit productive HAdV-F40 infection [59]. They also reported that the alternative splicing junction in this case does not follow the GT-AG splice consensus rule. Further research into cellular factors may be necessary to elucidate the molecular mechanism of HAdV-F40 alternative splicing.

Advancing research on alternative splicing (AS) in virus-infected cells is a key challenge. Regarding AS in virus-infected cells, in 1984, *E1a*-*E1b* overlapping mRNAs, which are not constitutively spliced, had been shown to be transcribed in the early stages of infection in HAdV-C2-infected KB cells [75]. Furthermore, as mentioned above, in 1991, Hashimoto et al. found *E1a*-*E1b* overlapping mRNAs in HAdV-F40-infected A549 cells, which are likely transcribed in cells capable of productive infection but not of abortive infection [59]. These mRNAs were from the earliest studies to suggest that AS is important for viral mRNA transcription.

Another curious phenomenon occurs during adenovirus replication within cells: the transition of the replication machinery from an early to a late state. At this point, it was presumed that the production of viral DNA-derived mRNA shifts from an early to a late state. This indicates that the cell progresses further toward viral control and acquires the virus’s unique viral mRNA splicing mechanism. The breakthrough in this research came in 1998, likely from the work of Kanopka et al. [87]. They demonstrated that a protein derived from an adenovirus late gene is involved in the regulation of late mRNA. Subsequent studies, including their findings, were reviewed by Biasiotto and Akusjärvi [88].

Furthermore, since the beginning of the 21st century, and particularly around 2020, numerous reports have emerged indicating that viral infections can significantly alter the splicing mechanisms within cells [89,90]. This is thought to occur when viral proteins and non-coding RNAs introduced into cells by viral infection alter host alternative splicing patterns, thereby forcing further splicing changes. A notable example is a report on what occurs when the coronavirus infects human cells. SARS-CoV-2 nonstructural protein 16 (Nsp16) hijacks splice site recognition mechanisms by binding to U1 and U2 snRNAs [91]. Such research is expected to become one of the new avenues for questioning what viral infection truly is.

### 5.4. E4 Region: Another Adenovirus Oncogene

**Figure 1:**
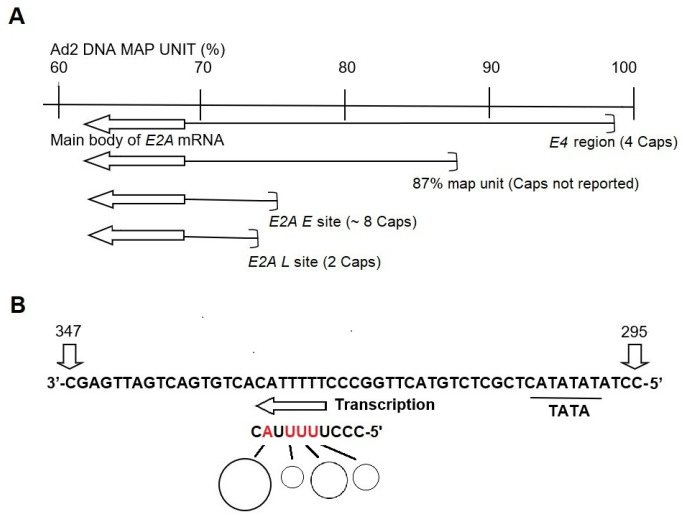
Location of HAdV-C2 early *E2* and *E4* mRNA 5′-terminal cap sequences on HAdV-C2 genome. (**A**) Alignment of *E2* mRNA transcription initiation sites. The physical map of 40% of HAdV-C2 genome is shown and is subdivided into 40 map units. There are four transcription initiation regions for *E2A* mRNA. Each region contains two or more cap sequences. The main body of the *E2* transcription is shown with arrows. (**B**) Alignment of 5′-terminal sequences of *E4* mRNA to HAdV-C2 DNA. The DNA sequence shown is nucleotide 295 to 347 from the righthand end of the r-strand [93,94]. There are four major transcription start sites, which are shown in red. The size of the circle represents the approximate relative strength of the transcription initiation. In addition, three more relatively weak 5′-terminal cap sequences were obtained in the *E4* mRNA [72].

Using the same method, Hashimoto et al. [72,73] and Kitani-Yasuda et al. [67] demonstrated that HAdV-C2 *E2* mRNA is transcribed using both the *E4* and *E2* promoters, as illustrated in Figure 1A of this review. Previous studies [72] have found that Ad2 *E4* mRNA has four major 5′-terminal sequences with unique base sequences, which have also been confirmed in the transcription initiation base sequences of *E2* mRNA [73]. Matsuo et al. obtained HAdV-C2 *E4* mRNA, performed in vitro translation to obtain proteins derived from the *E4* region and subsequently carried out peptide mapping [92]. Six proteins were identified: 35K, 23K, 22K, 21K, 18K and 11K. In the peptide mapping analysis, only the 11k protein differed from the others. This suggests that the mRNA encoding the 11K protein uses a different open reading frame to the others. In other words, it is thought that this mRNA has the potential for alternative splicing.

Furthermore, this protein has been shown to be immunoprecipitated with antibodies against HAdV-C2-transformed rat cells in HAdV-C2-infected cells [95]. Additionally, the HAdV-C5 E4 11K protein has been demonstrated to transform primary rat cells when the *E1a* gene is present [96]. However, these transformed cells did not necessarily retain the DNA that encodes the E1a and E4 11K proteins, as demonstrated by the researchers in a process known as “hit-and-run” transformation. It is thought that HAdV-C2 *E4* mRNA contains at least six distinct variants resulting from different splicing patterns. It is not yet known which of these variants exhibits the alternative splicing patterns arising from abnormal regulation. However, since *E4* mRNA can be obtained, it should be possible to create cDNA and determine the base sequence. The complete nucleotide sequence of the HAdV-C2 *E4* region has been determined [93,94].

As stated above, the transformation of cells by the *E4* domain occurs via a mechanism termed “hit-and-run”. However, an explanation for this mechanism has not yet been provided. The intracellular levels of HAdV-C2 *E1b* mRNA and the synthesis of the E1b 19K protein were compared in HAdV-C2-infected KB cells and KB18 cells (KB cells containing integrated HAdV-C2 *E1b* DNA) [97]. Looking at these results, the levels of *E1b* mRNA (22S and 13S) in transformed cells are 7% and 0%, respectively, compared to HAdV-C2-infected cells. Furthermore, in a similar comparison, the amount and synthesis of E1b 19K protein in transformed cells are 55% and 3% of infected cells, respectively [97]. Therefore, the mRNAs and proteins involved in cellular transformation may be significantly more stable than in virus-infected cells.

We still do not understand why the transcription start sites for HAdV-C2 early mRNA are so complex and numerous. However, it seems possible for us to infer how HAdV-C2 early mRNA came to possess such a complex TSS. This inference is based on a series of research findings by Berk’s group regarding the characteristics of other gene expression-promoting effects possessed by the HAdV-C2 E1a protein. They demonstrate that many different binding sites for TFs, including the *E1b* TATA box, a CREB/ATF binding site, and two E2F sites, can mediate E1a transactivation [98]. Ad E1a protein, a transactivation protein derived from the adenovirus oncogene, may be inducing unintended gene expression. It has been suggested that the mRNA transcription induced by the E1a protein may not only be adenovirus early mRNA, but also host cell mRNA [99]. Considering this, it may be necessary to broaden the scope of *E1a*+*E4* DNA transformation.

### 5.5. Advantages of Promoter Sharing Between E4 mRNA and E2 mRNA

Next, we will consider the advantages of *E4* and *E2* mRNA sharing the same promoter. What benefits does this promoter sharing offer for adenovirus replication? One possibility is that *E2* mRNA, which is the least expressed of the adenovirus early mRNAs, may be insufficient for viral replication [69,100]. How much is required for replication? The *E2a* and *E2b* mRNAs produce DBP, Ad Pol and pTP. DBP is an abundant protein because it is required to maintain the single-stranded nature of the parental strand for replication fork advancement. It is therefore possible that the *E4* promoter is being shared to increase expression levels. The 5′-terminal ends of the *E2* mRNA were mapped in at least four regions, as shown in Figure 1A. However, cap sequences have not been mapped in these regions. The cap information shown in Figure 1A was determined to be the result of separate primer extension analyses [67] and TSS analyses [72] using in vitro transcription initiation methods. The TSS of the *E2A L* was thought to be the site for late mRNA. However, the authors’ analysis showed that both TSSs are used for *E2* mRNA in the early stage of infection. In other words, the TSS of the *E2A L* TSS functions for both early and late mRNA. Additionally, they found that the transcription from *E2A L* is increased in the presence of CHX [67].

## 6. Concluding Remarks

In this review, we focused on how viruses use RNA and its modifications to facilitate replication and carcinogenesis in infected cells. We now understand that the *E1* and *E4* genes are adenovirus oncogenes. During the review process, we were most intrigued by the complexity of the TSS HAdV of early mRNA. The 5′-end heterogeneity of HAdV early mRNA has been demonstrated through other analytical methods. As discussed in Section 5.5 of this review, the 5′-end of HAdV-C2 *E2* mRNA is heterogeneous, as shown through primer extension analysis and TSS analysis. Furthermore, the 5′-end heterogeneity of HAdV-A12 *E1a* mRNA has been confirmed using the nuclease S1 mapping method [67,78]. The structural analysis of the TSS of HAdV-C2 early mRNA was reported by Baker and Ziff [100] using a method essentially identical to that described in [67,78]. However, they showed that a much smaller number of 5′-end-derived oligonucleotides were selected than those shown in this review. The methods used by these two groups to determine the 5′-terminus of each oligonucleotide separated by 2D fingerprinting and to select those with 2′-O-methylated nucleotides (pNms) at the 5′-terminus were different, and this difference is thought to be the reason for the differences in the results obtained.

Conversely, it has been suggested that mRNA with alternative splicing, which causes cancer, may be synthesized from genes with complex TSS [16]. Table 2 in this review shows the TSS of mRNA in the *E1* region, and Figure 1 shows those in the *E4* region. In addition, as described in each paper cited below, there are two 5′-end-derived oligonucleotides in *E1a* region mRNA [74] and three in *E4* region mRNA [72] whose complete base sequences have not yet been determined. Therefore, these two and three TSS derived oligonucleotides are not mapped to the vicinity of locations already indicated as *E1a* and *E4* TSS. We are interested in these five TSSs from the perspective of transcription regulation in the *E1* and *E4* regions.

The viral gene that attracted our attention the most was the structure of the oncogene of DNA tumor viruses and the modification of the corresponding mRNA. In adenoviruses, this corresponds to the *E1* region. Traditionally, the *E1* region has been considered to consist of two separate genes, *E1a* and *E1b*, which act in a coordinated manner. In this review, we present evidence that mRNAs sharing the *E1a* and *E1b* regions in HAdV-C2, HAdV-A12 and HAdV-F40 are transcribed by alternative splicing. We also explained how *E1a-E1b* cotranscripts are involved in cell transformation and tumorigenicity in HAdV-C2 and HAdV-A12, respectively. With regard to HAdV-F40, we discussed how the *E1a-E1b* cotranscript in human-derived cells can transform abortive infection into productive infection. Furthermore, modifications to the splicing mechanism within cells caused by viral infection are being discovered one after another. Given this, what relationship does this have with the complex TSS induction observed in HAdV?

In Section 5.4, we noted that in KB cells transformed cells with HAdV-C2 *E1b* region DNA, the level of mRNA expression is so low that conventional methods are difficult to detect. However, the protein is present at levels comparable to HAdV-C2-infected cells and functions adequately [97]. As described, the adenovirus cytocidal (cyt) mutant is a mutant virus containing mutation(s) in the 19K protein region of the adenovirus *E1b* DNA. The mutant viruses have been obtained from HAdV-C2, -C5, and -A12. These mutants exhibit cytotoxicity upon infection of KB or HeLa cells but do replicate. However, they are transformation-defective. In addition, the mutated E1b 19K protein has been shown to be degraded more rapidly than the wild-type 19K protein in infected cells. These experimental results suggest that the stability of the E1b 19K protein within the cell is crucial for the induction of cell transformation by HAdV. The intracellular stability of oncogene-derived proteins may be another important factor to consider when considering the mechanism of cell transformation by HAdV.

## Figures and Tables

**Table 1:** Products of RNase T2 digestion of HAdV-C2 early mRNA and pre-mRNA, and the chain length deduced from 5′-terminal cap.

	mono	di	tri	cap1	cap2	
Ribonucleotides	−2	−3	−4	−5	−6	Chain Length (nt)
mRNA [29,32]						
poly (A)^+^mRNA	99.55	-	-	0.2	0.25	1250
poly (A)^−^mRNA	99.2	0.11	0.13	0.27	0.3	960
pre-mRNA *						
poly (A)^+^pre-mRNA	99.63	0.26	0.02	0.07	0.02	5300
poly (A)^−^pre-mRNA	99.33	0.46	0.03	0.15	0.03	2900

*: Unpublished results. HAdV-C2-infected KB cells were labeled from 1 to 6 h post-infection with [^32^PO_4_] (1 mCi/mL) and methyl-[^3^H] methionine (200 μCi/mL) in the presence of cycloheximide [29,32]. To prevent contamination of pre-ribosomal RNA, pre-mRNA was purified in two-step purification procedures that involve poly(U)-Sepharose column chromatography and hybridization to Ad2 DNA.

**Table 2:** Adenovirus type 2 *E1* mRNA mapping of 5′-terminal RNase T1 oligonucleotides from fingerprinting analysis.

Spot Numbers of the 5′-Terminal Fragments		mRNA Hybridized with		
		*E1a* (0–4.5%)	*E1b* (4.5–8.0%)	8.0–10.7%
1	^32^P(m6)AmC(m)UCUUGp (Cap sequence of *E1a* mRNA)	+	+	+
2	^32^P(m6)AmC(m)AUCUGp (Cap sequence of *E1b* mRNA)	-	+	+
a	^32^P(m6) Am(2A, 2U) or (2C, 2U) or (A, C, 2U)Gp	+	+	+
b	^32^P(m6) Am(4A, 2U) or (3A, C, 2U) or (2A, 2C, 2U) or (A, 3C, 2U) or (4C, 2U)Gp	-	-	+
c	^32^P(m6) Am(2A, U) or (A, C, U) or (2C, U)Gp	-	-	+
d	^32^P(m6) Am(3A, U) or (2A, C, U) or (A, 2C, U) or (3C, U)Gp	+	+	+

+: 5′-terminal cap sequence shown left was detected in the mRNA hybridized with each of *E1* region. -: 5′-terminal cap sequence was not detected in the mRNA. Spots 1 and 2: complete sequences were determined [74]. Spots a to d: 5′-Terminal nucleotides (pNms) were determined through digestion with nuclease P1 and 2-D thin layer chromatography. 3′-Nucleotide (Gp) was determined through RNase T1 specificity. Nucleotide length and the nucleotide composition of each spot were derived from the migration of each spot in the fingerprint [71,74].

## Data Availability

The original contributions presented in this study are included in the article. Further inquiries can be directed to the corresponding authors.
